# Comprehensive Characterization of Structural, Electrical, and Mechanical Properties of Carbon Nanotube Yarns Produced by Various Spinning Methods

**DOI:** 10.3390/nano12040593

**Published:** 2022-02-10

**Authors:** Takayuki Watanabe, Satoshi Yamazaki, Satoshi Yamashita, Takumi Inaba, Shun Muroga, Takahiro Morimoto, Kazufumi Kobashi, Toshiya Okazaki

**Affiliations:** 1 CNT-Application Research Center, National Institute of Advanced Industrial Science and Technology, Tsukuba 305-8565, Japan; takayuki.watanabe.r@gmail.com (T.W.); takumi.inaba@aist.go.jp (T.I.); muroga-sh@aist.go.jp (S.M.); t-morimoto@aist.go.jp (T.M.); kobashi-kazufumi@aist.go.jp (K.K.); 2 Research Association of High-Throughput Design and Development for Advanced Functional Materials (ADMAT), Tsukuba 305-8565, Japan; satoshi.mayama.yamazaki@furukawaelectric.com (S.Y.); satoshi.yamashita@furukawaelectric.com (S.Y.)

**Keywords:** carbon nanotubes, yarn, electrical conductivity, tensile strength, nanotube length

## Abstract

A comprehensive characterization of various carbon nanotube (CNT) yarns provides insight for producing high-performance CNT yarns as well as a useful guide to select the proper yarn for a specific application. Herein we systematically investigate the correlations between the physical properties of six CNT yarns produced by three spinning methods, and their structures and the properties of the constituent CNTs. The electrical conductivity increases in all yarns regardless of the spinning method as the effective length of the constituent CNTs and the density of the yarns increase. On the other hand, the tensile strength shows a much stronger dependence on the packing density of the yarns than the CNT effective length, indicating the relative importance of the interfacial interaction. The contribution of each physical parameter to the yarn properties are quantitatively analyzed by partial least square regression.

## 1. Introduction

Carbon nanotubes (CNTs) display very high electrical conductivities, thermal conductivities, as well as mechanical strengths, and sorption abilities [1,2,3,4,5]. Because they also have lower densities than metals such as copper and steel [6], CNTs have potential as alternating-current power cables and wires. In fact, their estimated electrical conductivity is as high as 900,000 S/cm, which is much larger than that of copper (600,000 S/cm) [7]. Additionally, the tensile strength of CNT bundles is ~80 GPa, whereas that of steel is ~1 GPa [2].

To preserve superior properties in macroscopic CNT-based structures, the production of yarns composed of CNTs offers a potential for high-strength and lightweight materials that are also thermally and electrically conductive [8,9,10,11,12]. Macroscopic CNT yarns show an electrical conductivity and tensile strength of 10,900 S/cm and 9.6 GPa, respectively [13,14,15]. Their values are within a factor of those of aluminum and commercially available carbon fibers, respectively. CNT yarns exhibit a strength similar to carbon fibers and an electrical conductivity similar to metal.

**Scheme 1:**
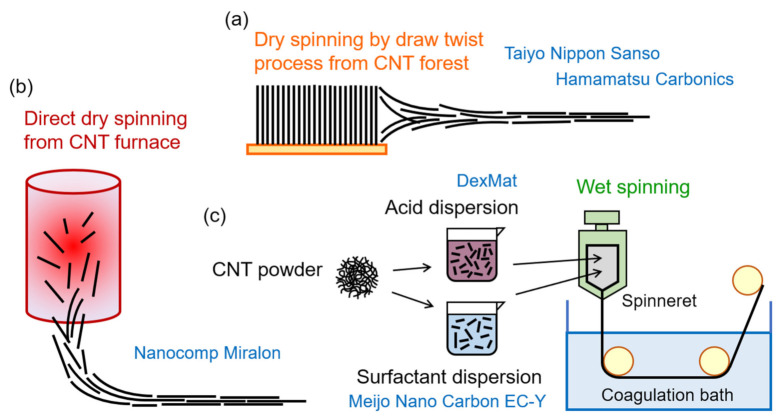
Schematic representation of spinning methods for CNTs. (**a**) Dry spinning by draw twist process from CNT forest, (**b**) dry spinning from CNT furnace, and (**c**) wet-spinning.

Currently, CNT yarns are mainly produced by three methods: dry spinning from multi-walled CNT (MWCNT) forests, direct spinning from a CVD furnace, and wet-spinning of CNTs (Scheme 1). Elucidating their structures, properties, and relationships are critical to realize practical applications of CNT yarns.

We previously investigated the electrical and mechanical properties of wet-spun CNT yarns using far-infrared (FIR) spectroscopy. These properties are related to the effective CNT length [16,17,18]. The observed FIR peak can be explained by the one-dimensional plasmon model. Consequently, the estimated length can be ascribed to that of the clean and straight CNT portion between defects or kinks (effective CNT length) [19,20,21,22]. FIR spectroscopy is applicable to single-walled CNTs (SWCNTs) and MWCNTs because the resonant frequency is insensitive to the CNT diameter [19,21]. On the other hand, the G-band/D-band ratio in the Raman spectrum strongly depends on the CNT diameter and CNT types.

Here, we systematically investigate the structure of various CNT yarns and the properties of the constituent CNTs. Our study evaluated yarns fabricated by three different spinning methods using various CNTs. We observed the yarn structures and cross-sections by scanning electron microscopy (SEM). We also estimated the average CNT diameters and wall numbers by transmission electron microscopy (TEM), the CNT alignment by wide angle X-ray diffraction (WAXD), the G/D ratios and the radial breathing modes by Raman spectroscopy, and the effective CNT lengths by FIR spectroscopy.

Then we evaluated the electrical conductivities and the tensile strengths of the yarns, and their correlation with the yarn and CNT properties. The electrical conductivity of the yarns is significantly correlated with the effective CNT length, yarn density, and CNT alignment in the yarns. In contrast, the tensile strength depends more on the yarn density and the CNT alignment. To quantitatively evaluate the contribution of each physical parameter to the properties of CNT yarn, the measurement data was analyzed using the partial least square (PLS) regression [23,24,25].

## 2. Materials and Methods

The six commercially available CNT yarns were studied here (Scheme 1). Two CNT yarns by dry spinning from CNT forests were purchased from Hamamatsu Carbonics Corporation (Shizuoka, Japan) and Taiyo Nippon Sanso Corporation (Tokyo, Japan). The CNT yarn by direct spinning from a CVD furnace (Miralon CNT yarn) was obtained from Nanocomp Technologies Inc. (Merrimack, NH, USA). The wet-spinning CNT yarns from surfactant-assisted aqueous dispersion and strong acid dispersion were purchased from Meijo Nano Carbon Co., Ltd. (Aichi, Japan) (EC-Y type I and II) and DexMat Inc. (Houston, TX, USA), respectively.

The morphologies of the CNT yarns were observed by SEM microscopy (Hitachi SU-8200, Tokyo, Japan). The diameter and wall number of the CNTs were estimated by TEM microscopy (EM002B, Topcon, Tokyo, Japan), while WAXD (AichiSR, beam line BL8S3, Aichi, Japan) assessed the CNT alignment in the yarns. The photon energy was 13.48 KeV (λ = 0.092 nm). The sample-to-detector distance was 0.208 m. The WAXD spectra were measured using a two-dimensional detector (R-AXIS VII++). The scattering wave vector q = 4πsinθ/λ, where θ is the scattering angle. The Raman spectra were measured with inVia (Renishaw, Wotton-under-Edge, England, UK) with an excitation wavelength of 532 nm. The effective length of each CNT was estimated from its FIR optical absorption spectrum [20,22]. The FIR measurements were acquired using Vertex 80v (Bruker Optics, Billerica, MA, USA) and TR-1000 (Otsuka Electronics, Osaka, Japan). The average structural CNT lengths were estimated from the AFM measurements (Shimadzu SFT-4500, Kyoto, Japan). 

The electrical resistance of the CNT yarn was measured using the four-terminal method with a probe system (Summit12000, Cascade Microtech, Beaverton, OR, USA) and a semiconductor device analyzer (B1500A, Keysight, Santa Rosa, CA, USA). Three samples were measured for each CNT yarn type. The distance between the two terminals of the differential voltage measurement system was 30 mm. The obtained values were averaged for, at least, three samples for each CNT yarn.

The breaking strengths of the CNT yarns were measured in tensile tests with a micro strain tester (MST-I, Shimadzu, Kyoto, Japan). The test pieces and the test conditions followed the Japanese standard, JIS R 7606, which is a standard protocol for carbon fiber tensile testing. The gauge length was fixed to 25 mm, and the head speed was fixed to 1 mm/min. Three samples were measured for each CNT yarn type.

Thermogravimetric analysis (TGA) was used to estimate the carbonaceous purity of the CNT yarns with TGAQ500 (TA instruments, New Castle, DE, USA).

PLS models were constructed from the covariance between the standardized response variables (electrical conductivity or tensile strength) and the standardized explanatory variables (physical parameters). Details for select measurement procedures are described in the Supplementary Materials.

## 3. Results and Discussion

### 3.1. Structural Characterizations of Commercially Available CNT Yarns and Their Constituent CNTs

**Figure 1:**
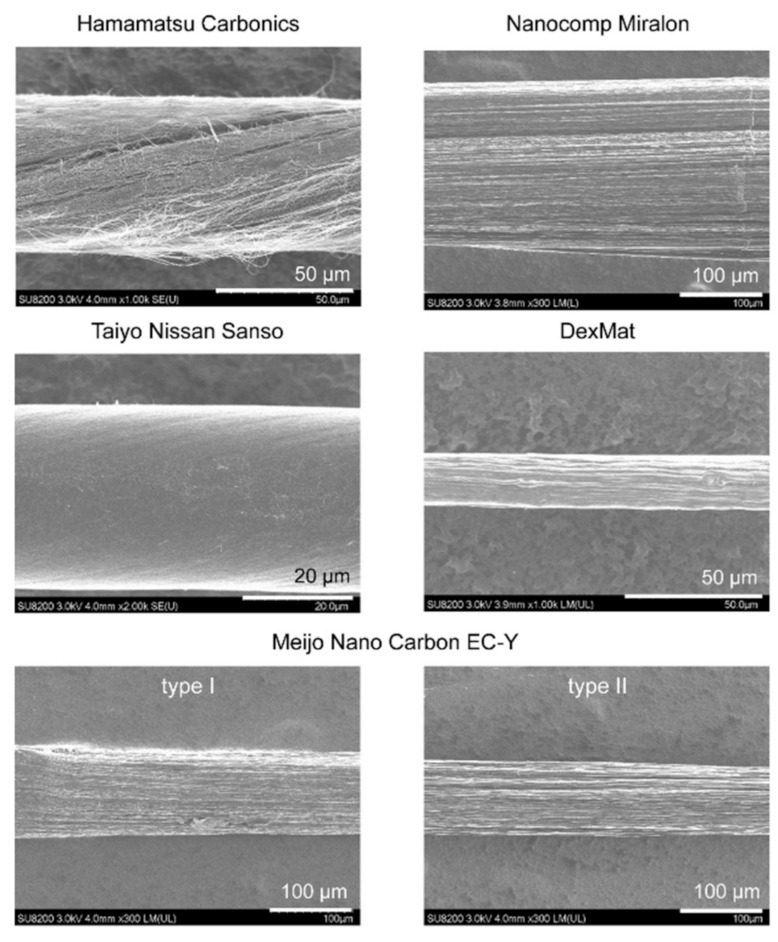
Side-view SEM images of CNT yarns.

**Figure 2:**
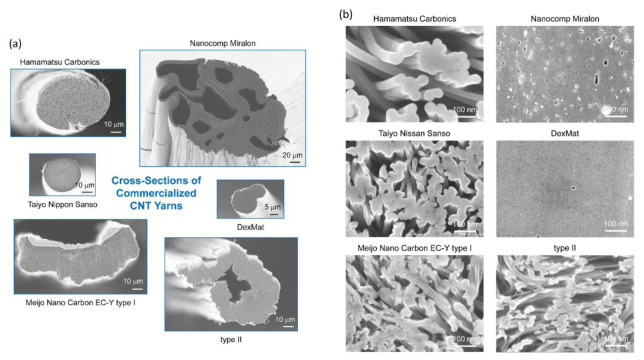
Cross-sectional SEM images of CNT yarns. (**a**) Low-magnification images. (**b**) High-magnification images.

The structural and physical properties of CNT yarn strongly depend on the spinning method [8,9,10,11,12,26]. Figure 1 and Figure 2 show SEM images of the CNT yarns evaluated in this study. Hamamatsu and Taiyo Nippon Sanso yarns were made by dry spinning from CNT forests [27]. Their estimated diameters by laser micrometer are 59 and 31 μm, respectively (Table 1, see the Supporting Information). This method spins CNTs from a CNT forest by twisting. The twisted structure is visible from the side views (Figure 1). The roundness of the cross-sections exceeds those of the other yarns, reflecting the high controllability of the dry spinning methods (Figure 2a). The network structures in the high-magnification images show the different diameters of the constituent CNTs (Figure S1 in the Supporting Information).

Nanocomp Miralon yarn is produced by direct spinning from a CVD furnace [28]. It has a unique structure among the CNT yarns, including conventional direct spun CNT yarns [29,30,31]. The low-magnification image shows non-negligible voids in the yarn (Figure 2a), whereas the CNTs seem well packed and sub micrometer voids rarely appear in the high-magnification image (Figure 2b). The estimated yarn diameter is 230 μm, which is the largest among the yarns in this study (Table 1).

Meijo CNT yarns are wet-spun in a surfactant-assisted aqueous dispersion [32]. These yarns have a ribbon shape, and the CNT bundles are sparsely entangled, rather than aligned (Figure 2). DexMat yarn is produced by wet-spinning CNTs dispersed in a strong acid [10]. DexMat yarn has a rounded cross-section, and CNTs are well packed similar to Nanocomp Miralon (Figure 2). Despite its high density indicated by the cross-section, the side view clearly shows a bundled structure (Figure S1). DexMat has thicker bundles but its average diameter is smaller (20 μm) than those of Meijo yarns (90 μm and 76 μm for type I and type II, respectively) (Table 1).

**Figure 3:**
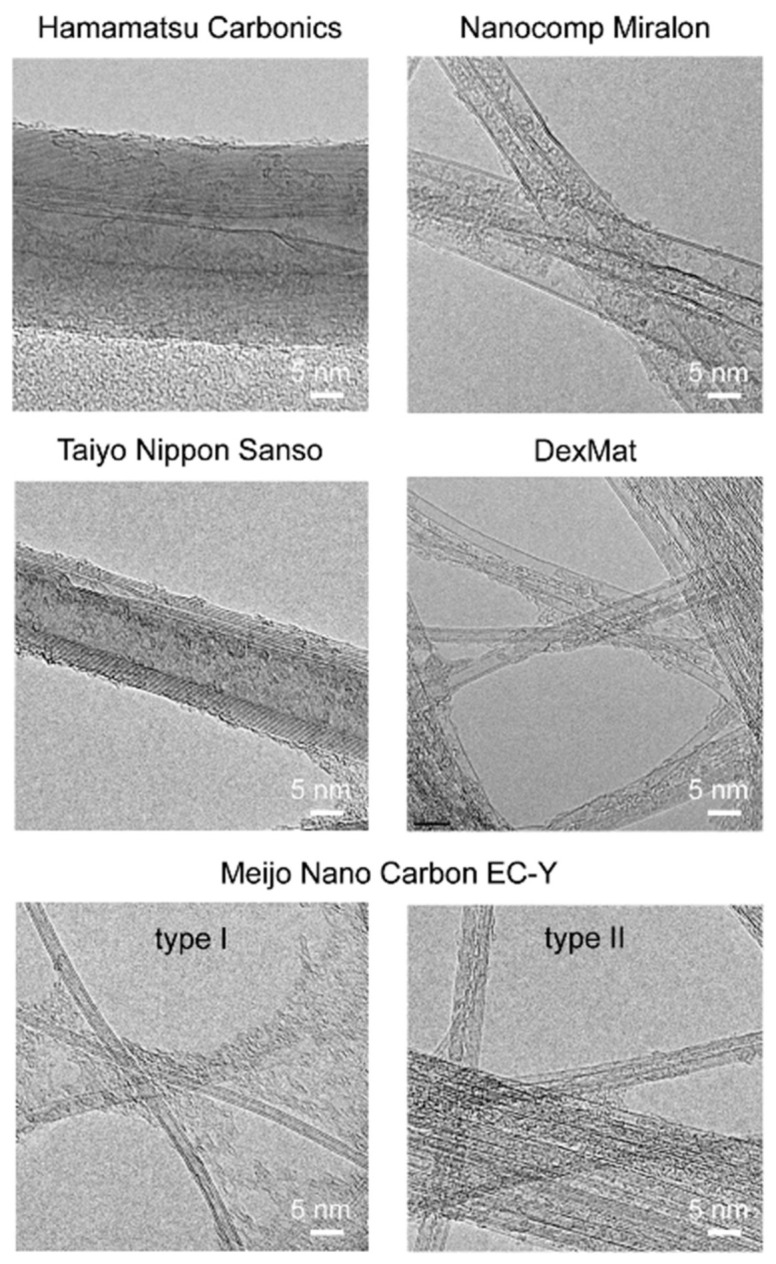
TEM images of constituent CNTs of each yarn.

The diameters and the wall numbers of the constituent CNTs were estimated from TEM observations (Figure 3 and Figure S2, Table 1). The average values of the CNT diameters are widely distributed from 1.4 to 45 (Figure S2). Based on the observed wall numbers, Hamamatsu Carbonics and Taiyo Nippon Sanso yarns consist of MWCNTs. The other yarns, Nanocomp Miralon, Meijo, and DexMat, contain both FW and SWCNTs.

**Figure 4:**
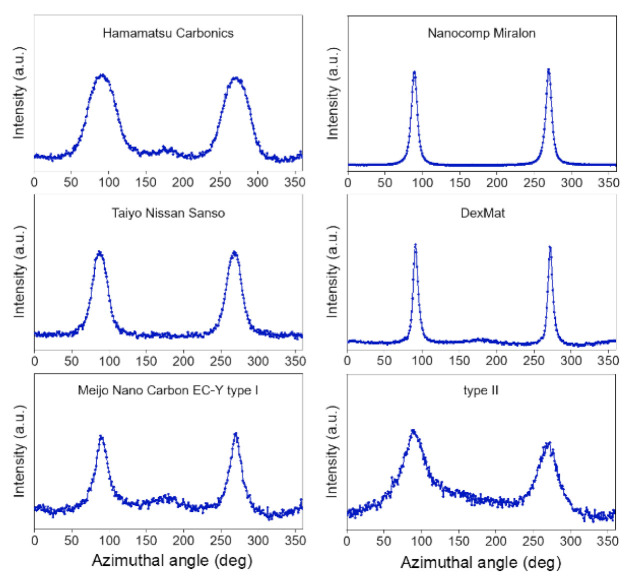
WAXD azimuthal scan of CNT yarns.

The CNT alignment along the long axis of the yarns was estimated by WAXD. The WAXD spectra showed a broad peak between *q* = 11.41 and 25.27 nm^−1^, which corresponds to the planes perpendicular to the CNT axis. The CNT alignments were estimated based on the full width at half maximum (FWHM) of the azimuthal scan of the peaks (Figure 4). The Hamamatsu and Meijo yarns have Herman orientation factors of 0.78–0.86 (Table 1). On the other hand, DexMat and Nanocomp yarns display higher alignments (=0.94). Note that the broad small peak at an azimuth angle = 150–200° is X-ray scattering from the Kapton tape, which is used as a window material for the 2D detector and x-ray guideline.

### 3.2. Spectroscopic Characterizations

**Figure 5:**
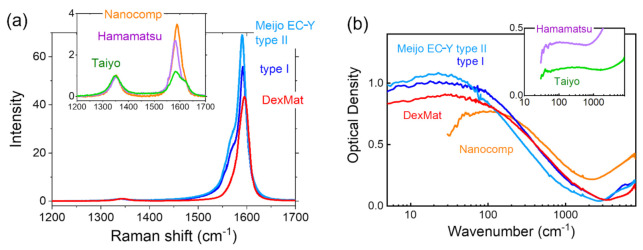
(**a**) Resonance Raman spectra and (**b**) far-infrared spectra of the constituent CNTs in yarns.

We performed two spectroscopic characterizations (Figure 5). Resonance Raman spectroscopy is a powerful tool for evaluating CNT-based materials (Figure 5a). The integrated G/D intensity ratio of each yarn ranges from 1.2 to 98. Meijo and DexMat tubes show relatively larger G/D values, whereas the Taiyo Nippon Sanso, Hamamatsu Carbonics, and Nanocomp Miralon samples show smaller values around two (Table 1).

Another parameter is the effective length (*L*_eff_), which is estimated from the optical absorption of CNTs in the FIR region (Figure 5b) [19,20,21,22]. Here, the length estimated based on a one-dimensional plasmon resonance model [22] corresponds to the length (or the size) in the high-crystallinity region of the CNT. Therefore, *L*_eff_ directly correlates with the physical properties of the CNT films and wet-spun yarns such as electrical conductivity [16,19]. SW and FWCNTs have a longer *L*_eff_, whereas MWCNTs show a shorter *L*_eff_ (Table 1).

### 3.3. Electrical Conductivity

**Figure 6:**
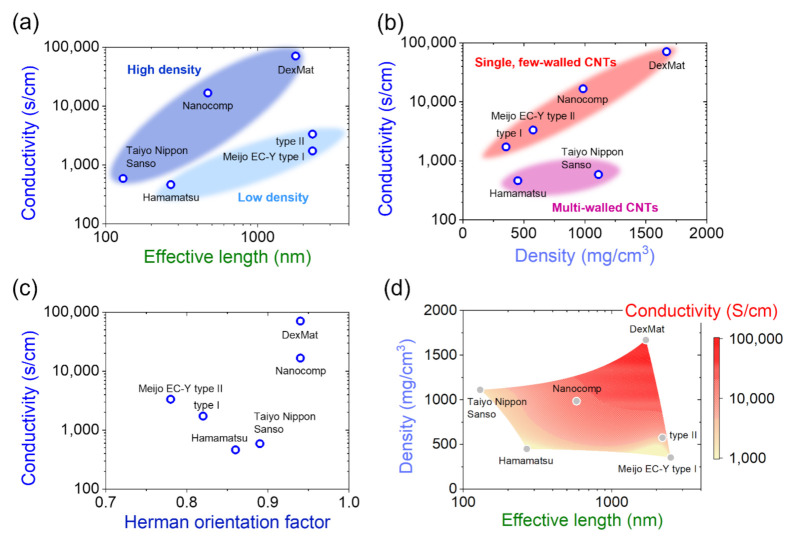
(**a**) Effective length, (**b**) density, and (**c**) Herman orientation factor dependences of the electrical conductivity of yarns. (**d**) 2D contour plot of the yarn conductivity.

Figure 6a plots the electrical conductivities of the yarns as functions of *L*_eff_ of the constituent CNTs. The obtained values are typical for the given spinning method [14,16,17,18,26,28]. The values range over almost two orders of magnitude. There is no apparent correlation between electrical conductivity and the effective length. However, dividing the CNT yarns into two groups by their density (i.e., high and low densities) reveals a positive dependence on the effective length within the group.

The density dependence of the electrical conductivity does not show a clear trend (Figure 6b). Similar to above, classifying the CNT yarns into two groups depending on the number of walls, (i.e., MWCNTs and FW/SWCNTs) highlights an obvious dependence on the yarn density. Since the wall number of CNTs is usually related to the crystallinity [21], the effective lengths of FW and SWCNTs are longer than those of MWCNTs. Consequently, the electrical conductivity depends on both *L*_eff_ (crystallinity) and the yarn density (Figure 6d). If one of these factors is low, the electrical conductivity will be low. In other words, both the CNT effective length and density must be controlled to improve the conductivity of CNT yarn.

The Herman orientation factor dependence of the electrical conductivity (Figure 6c) is similar to the density dependence (Figure 6b). This is reasonable since highly aligned yarns have larger densities (Figure 1 and Figure 2). Indeed, the calculated correlation coefficient between the Herman orientation factors and the yarn densities is 0.78.

Since *L*_eff_ represents the length of the high-crystallinity region of CNTs, CNTs with a longer *L*_eff_ should exhibit a higher conductivity because there are fewer CNT junctions per unit length. On the other hand, a common method to estimate the CNT length is performing counting experiments with atomic force microscopy (AFM) [19,33,34]. Since the length estimated by the AFM observation corresponds to the physically and structurally connected one, we designated it as the ‘structural length’ (*L*_str_) in this paper. As shown in Figure S4a in the Supporting Information, *L*_eff_ and *L*_str_ do not show a clear correlation, which is consistent with the previous report [19]. Figure S4b shows the electrical conductivity of the yarns as a function of *L*_str_ estimated from the AFM observations. The electrical conductivity of Hamamatsu Carbonics yarn is too low for the large *L*_str_. This means that although the CNTs are physically connected, defects or kinks on the tube wall cause substantial electrical resistance.

### 3.4. Tensile Strength

**Figure 7:**
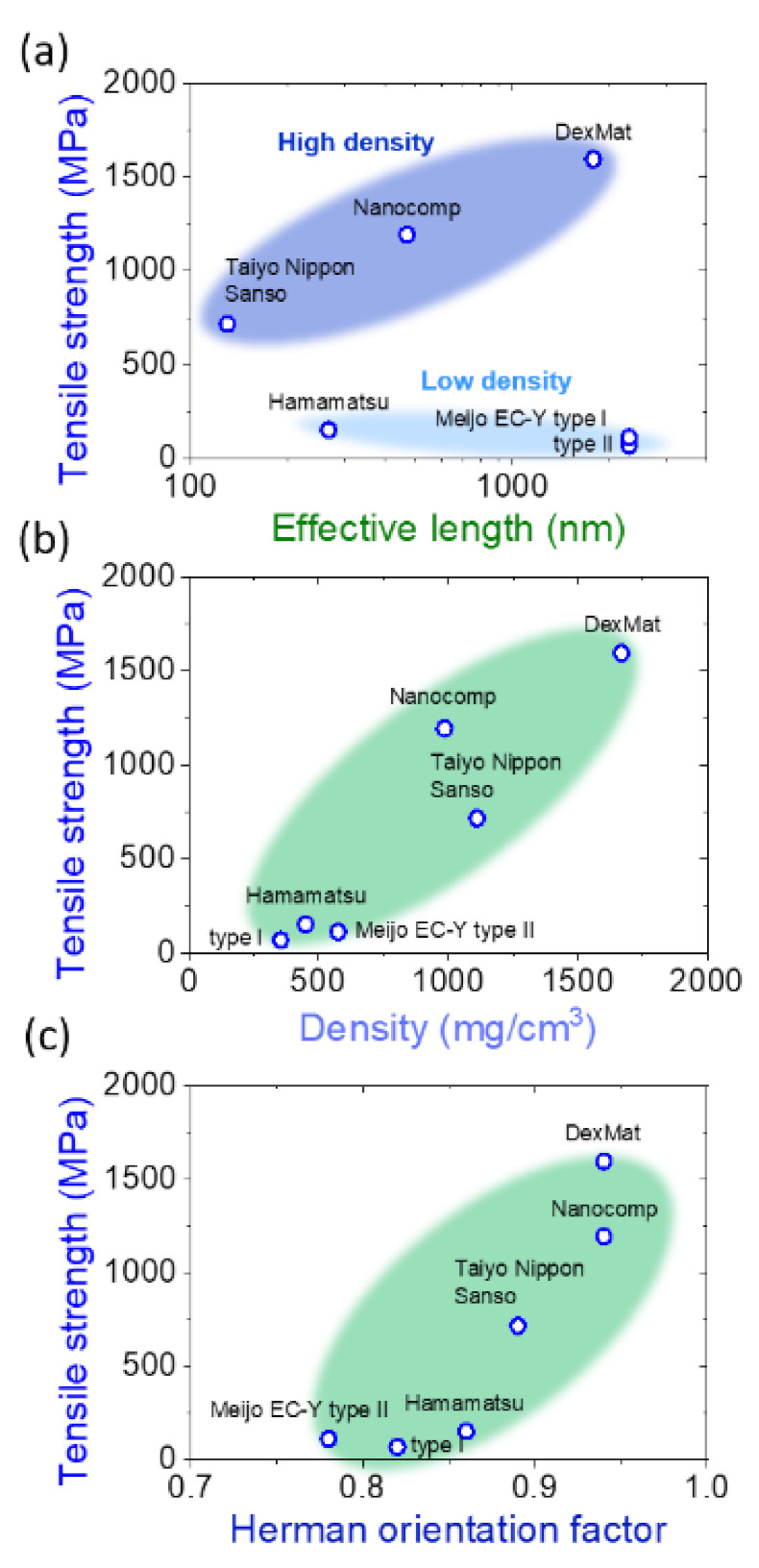
(**a**) Effective length, (**b**) density, and (**c**) Herman orientation factor dependences of the tensile strength of yarns.

Figure 7a,b show the tensile strengths of CNT yarns as functions of *L*_eff_ and the yarn density. The obtained tensile strengths are typical values for CNT yarns produced by the corresponding spinning method [14,16,17,18,26,27,28,35,36]. Similar to the conductivity, we classified the results into two groups by the yarn density. Although high-density yarns seem to depend on *L*_eff_, low-density yarns do not. This means that *L*_eff_ is a good parameter for the tensile strength only if the yarn is sufficiently dense.

Compared with the electrical conductivity, the tensile strength shows a stronger dependence on the yarn density (Figure 7b) and Herman orientation factor (Figure 7c). Intuitively, CNTs in a dense yarn should have a larger total contact area with neighboring CNTs than those in a low-density yarn. Until the individual CNTs start to slip relative to each other, a greater frictional force is applied to the CNT surface. Thus, dense yarn with a high alignment exhibits a higher tensile strength. Apparently, the increase in friction between CNTs should be higher for longer CNTs. This situation is consistent for CNT yarns with a higher density (Figure 7a).

### 3.5. PLS Regression Analysis

**Figure 8:**
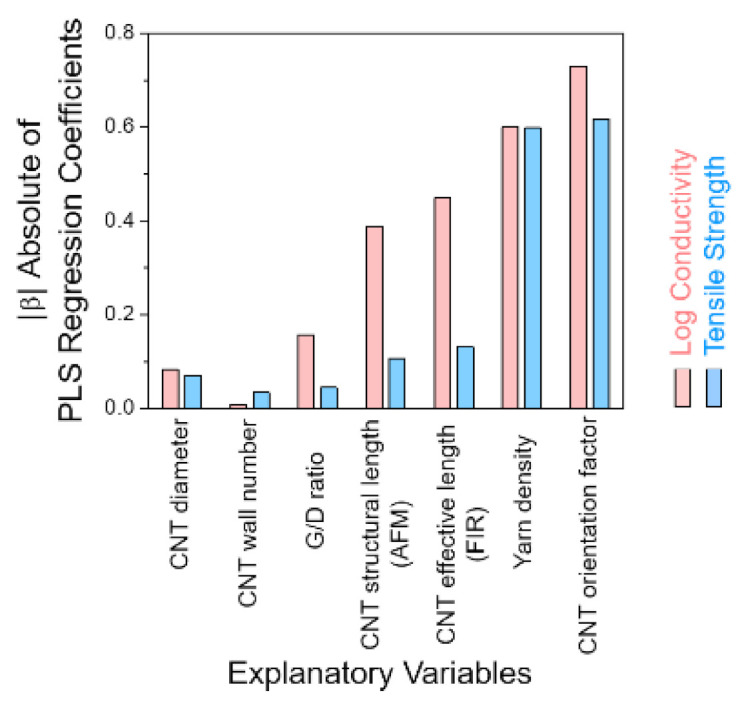
PLS analysis of the electrical conductivity and tensile strength of CNT yarns.

The contribution of each physical parameter to the electrical conductivity and the tensile strength was quantitatively analyzed by PLS regression. PLS regression is commonplace in statistics or machine learning, especially for spectroscopic data with high multi-dimensional collinearity [23,24,25]. PLS regression can efficiently extract information from data even with the multi-dimensional collinearity of variables. Since the absolute values of the regression coefficients of standardized variables (|*β*|) are related to the weights/importance, they are good indicators for interpreting or refining the variables. Based on the results (Figure 8), the contributions of the effective length, yarn density, and degree of orientation are higher than the others for the electrical conductivity. This may indicate that it is important to form a current path with the shortest distance without CNT-CNT junctions in CNT yarns.

On the other hand, the tensile strength is less dependent on the effective length and the G/D ratio, which are parameters of CNT quality. The density and the Herman orientation factor contribute almost equally. The fracture mechanism of CNT yarn in tension should be dominated by the withdrawal of CNT. Thus, how close a CNT is to other CNTs is more important than the strength of an individual CNT.

## 4. Conclusions

We here conducted comprehensive characterizations of six CNT yarns produced by the different spinning methods with various CNTs. The structural properties of the yarns were investigated by SEM, laser microscope and WAXD. The properties of the constitute CNTs such as diameter, wall number, and effective length were characterized by TEM, resonance Raman, and FIR spectroscopy. The relationship between the physical properties of the yarns and the obtained structural parameters were then examined. The CNT effective length and the yarn density well characterize the electrical conductivity of all CNT yarns. On the other hand, the tensile strength of the yarns exhibits much stronger dependence on the yarn density. The stronger correlation of the tensile strength to the yarn density indicates the importance of the interfacial interaction with adjacent CNTs (or CNT bundles) in determining the mechanical properties. PLS analysis can explain the above observations quantitatively. The present study offers the scientific perspectives on the neat CNT yarns. One of the important findings is that DexMat yarn is composed of the longest class of CNTs that we have ever measured [16,17,18,19,20], and the packing density is close to the highest limit [6]. Therefore, to improve the properties of the CNT neat yarn further, post-treatment processes such as doping [10] and cross-linking [37] will become crucial. The doping effects on the electronic structures and the electrical conductivities of DexMat CNT yarn have been investigated by the present research group, which will appear in near future.

## Figures and Tables

**Table 1:** Physical properties of the CNT yarns in this study.

CNT Yarns	Hamamatsu Carbonics	Taiyo Nippon Sanso	Nanocomp Miralon	Meijo EC-Y Type I	Meijo EC-Y Type II	DexMat
Yarn diameter (μm)	59	31	230	90	76	20
Average diameter of CNTs (nm)	45 ± 1	12 ± 0.2	5.3 ± 0.1	1.8 ± 0.05	1.7 ± 0.04	2.2 ± 0.05
Average wall number	50 ± 2	8.5 ± 0.1	1.9 ± 0.03	1.4 ± 0.05	1.4 ± 0.05	1.6 ± 0.05
CNT type	MWCNT	MWCNT	FWCNT	SWCNT	SWCNT	FWCNT
WAXD (002) FWHM (degree)	42.8	23.7	9.4	21.4	38.4	7.7
Herman orientation factor	0.86	0.89	0.94	0.82	0.78	0.94
G/D ratio	2.2 ± 0.03	1.2 ± 0.03	3.3 ± 0.3	50 ± 5	98 ± 9	31 ± 2
CNT effective length (nm)	270	130	470	2300	2300	1800
CNT length by AFM (μm)	11 ± 2 ^(a)^	1.1 ± 0.07 ^(a)^	3.9 ± 0.4 ^(b)^	3.2 ± 0.2 ^(b)^	1.7 ± 0.1 ^(b)^	1.3 ± 0.1 ^(b)^
Yarn Density (mg/cm^3^)	450	1111	985	353	575	1670
Electrical conductivity (S/cm)	462	588	16,627	1725	3334	70,659
Tensile strength (MPa)	151	716	1193	69	111	1595

^(a)^ Individual MWCNTs; ^(b)^ Bundles of SW or FWCNTs.

## Data Availability

Not applicable.

## Supplementary Materials

The following supporting information can be downloaded at: https://www.mdpi.com/article/10.3390/nano12040593/s1, Figure S1: Side-view SEM images of CNT yarn at high magnification; Figure S2: (a) TEM images of CNTs used in each yarn. (b) Histograms of CNT diameter and wall number; Figure S3: (a) AFM images of each CNT. (b) Histograms of CNT length; Figure S4: (a) Relationship between structural length and effective length. (b) Structural length dependences of electrical conductivity; Figure S5: (a) Photograph of custom-made fiber diameter measurement system including high-speed optical micrometer (LS-9006MR series, Keyence). (b) Schematic illustration of how to measure yarn diameter. (c) Cross-sectional SEM image of Nanocomp Miralon yarn. (d) Voids extracted from the SEM image by using ImageJ software; Table S1: Carbonaceous content of CNT yarns estimated by TGA.
