# Supplementary material for: Comprehensive Characterization of Structural, Electrical, and Mechanical Properties of Carbon Nanotube Yarns Produced by Various Spinning Methods
Source: Nanomaterials (Basel). 2022 Feb 10;12(4):593. doi: 10.3390/nano12040593 (PMC8880327; doi:10.3390/nano12040593)
Supplement: Supplementary file 1 [file nanomaterials-12-00593-s001.zip › nanomaterials-1574293-supplementary.pdf]

Supporting Information

Comprehensive characterization of structural, electrical, and mechanical properties of carbon nanotube yarns produced by various spinning methods

Takayuki Watanabe[1], Satoshi Yamazaki[2], Satoshi Yamashita[2], Takumi Inaba[1], Shun Muroga[1], Takahiro Morimoto[1], Kazufumi Kobashi[1] and Toshiya Okazaki[1,*]

[†]CNT-Application Research Center, National Institute of Advanced Industrial Science and Technology (AIST), Tsukuba, 305-8565, Japan
[‡]Research Association of High-Throughput Design and Development for Advanced Functional Materials (ADMAT), Tsukuba, 305-8565, Japan

*email: toshi.okazaki@aist.go.jp

Side-view SEM images of CNT yarn at high magnification

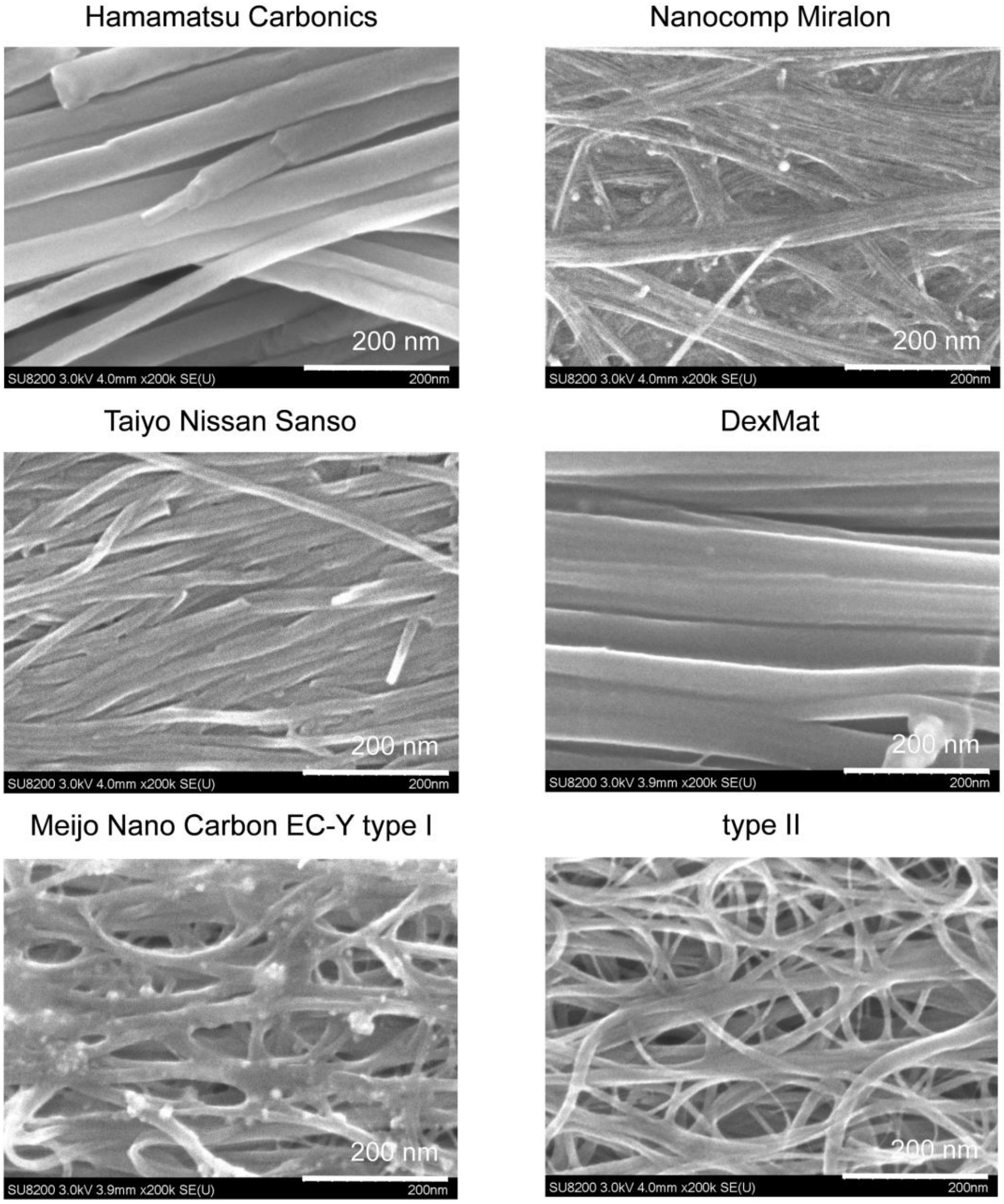

Figure S1. Side-view SEM images of CNT yarn at high magnification.

Procedure of TEM observations

The diameter and wall number of the CNTs were estimated by TEM microscopy (EM002B, Topcon, Tokyo, Japan). Sample grids were fabricated as follows. 0.5 mg of CNT was dispersed into 20 ml of 1,2-Dichloroethane by sonication (VCX130, Sonics & Materials Inc. (Newtown, CT, USA), power amplitude of 40% and pulse mode (1 s on, 1 s off)). The processing time was more than 4 hours. Droplets of CNT dispersion were cast on the TEM grid. Figure S2a shows typical TEM images, and

Fig. S2b shows histograms of the diameter and wall number of the constituent CNTs. Compared with the as-received CNT powders, relatively larger deposits can be seen depending on yarn type. This is because CNT yarn sometimes includes surfactants, dopants, and other materials that persist after the processing.

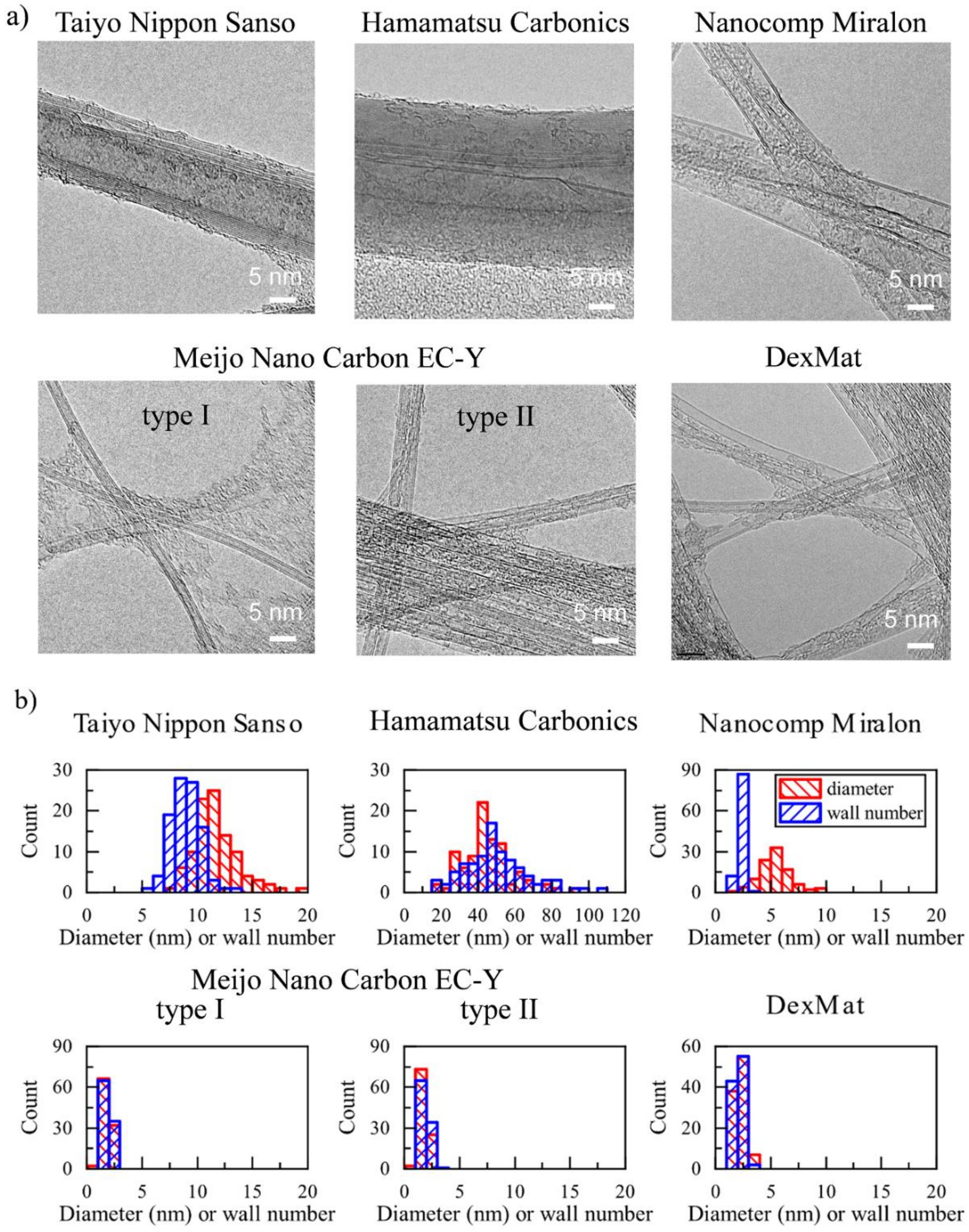

Figure S2. a) TEM images of CNTs used in each yarn. b) Histograms of CNT diameter (red) and wall number (blue).

Measurement of CNT effective length

The effective length of each CNT was estimated from the far-infrared (FIR) optical absorption spectrum (Fig. 5b). The details of the measurement procedure are described elsewhere.[1] Briefly, CNT thin film (so-called 'buckypaper') was made from CNT dispersions by sonicating CNT yarn. Each CNT dispersion was made from approximately 0.5 or 1 mg of CNT yarn and 20 ml deionized water with a surfactant. Sodium dodecylbenzene sulfonate (SDBS) was used as the surfactant, and its weight density was adjusted to 1 wt%. The mixtures were mildly dispersed with a magnetic stirrer at around 600 rpm for more than 3 days. Subsequently, a bath-type sonicator was used for dispersion. The frequency was 45 kHz, and the sonication time ranged from 4 minutes to 1 hour depending on the yarn dissolution. 700 to 2500 ul of the dispersions were used to make each piece of buckypaper. A Vertex 80v (Bruker Optics, Billerica, MA, USA) and TR-1000 (Otsuka Electronics, Osaka, Japan) were used for the FIR measurements.

Generally, sonication process causes the shortening in CNT length. Hence, in the present experiment, we carefully disentangle CNT yarns by the bath-sonication as short duration as possible. For example, it was estimated that the effective length of CNTs in Meijo yarns was ~2300 nm and the electric conductivities of the yarns were around 2000 S/cm (see Table 1 and Fig. 6a). The relationship between the effective length and the conductivity is consistent with the previous yarns that were produced by the same Meijo CNTs and the same wet-spinning method, in which the effective lengths were measured before spinning of the yarns.[2] The coincidence suggests that the present bath-sonication doesn't affect severe effects on the effective length. Hence, we believe that our main conclusion doesn't change very much by the bath-sonication processes.

Measurement of CNT structural length

The average CNT lengths were estimated from AFM measurements. The CNT dispersions used for the FIR spectroscopy were used for these measurements. The dispersions were cast on Si substrate and rinsed sufficiently in water. AFM measurements were conducted with an SFT-4500 (Shimadzu, Kyoto, Japan). Figure S3a and b show typical AFM images of the CNTs and histograms of the counted lengths, respectively. According to the shape of the fiber in the AFM images and the comparison of the height determined by AFM and the CNT diameter by TEM, the Hamamatsu and Taiyo Nippon Sanso CNTs were almost de-bundled and the measured lengths therefore corresponded to lengths of individual CNTs. In contrast, the lengths corresponded to those of CNT bundles for the Nanocomp, Meijo and DexMat samples. The relationship between the structural and the effective lengths is shown in Fig. S4.

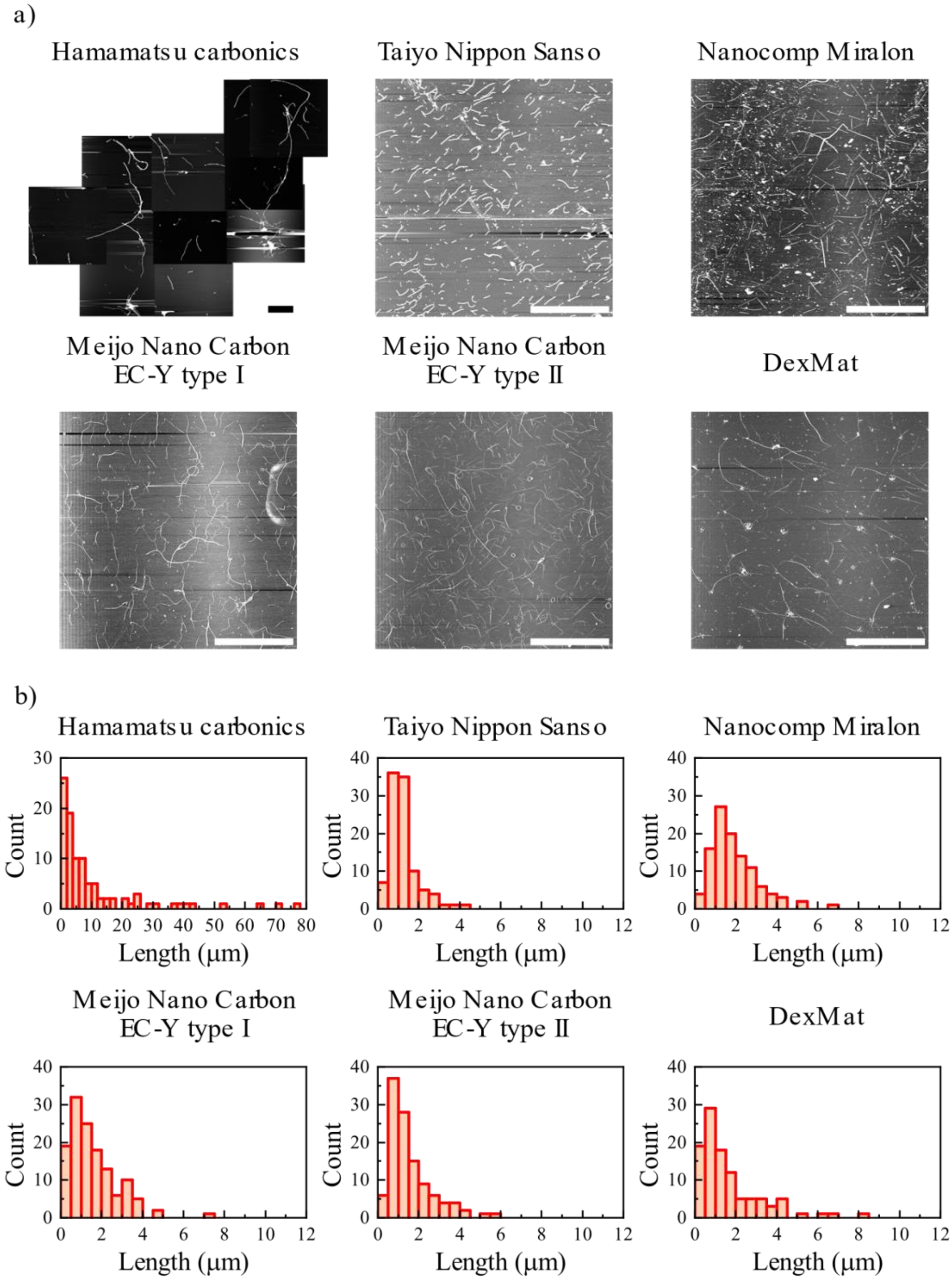

Figure S3. a) AFM images of each CNT. Scale bars correspond to 10 μm. Because Hamamatsu Carbonics CNTs are longer than the maximum scanning size (30 μm), the CNT length was measured by merging several images. b) Histograms of CNT length. Note that CNTs of Nanocomp, Meijo and DexMat are bundled.

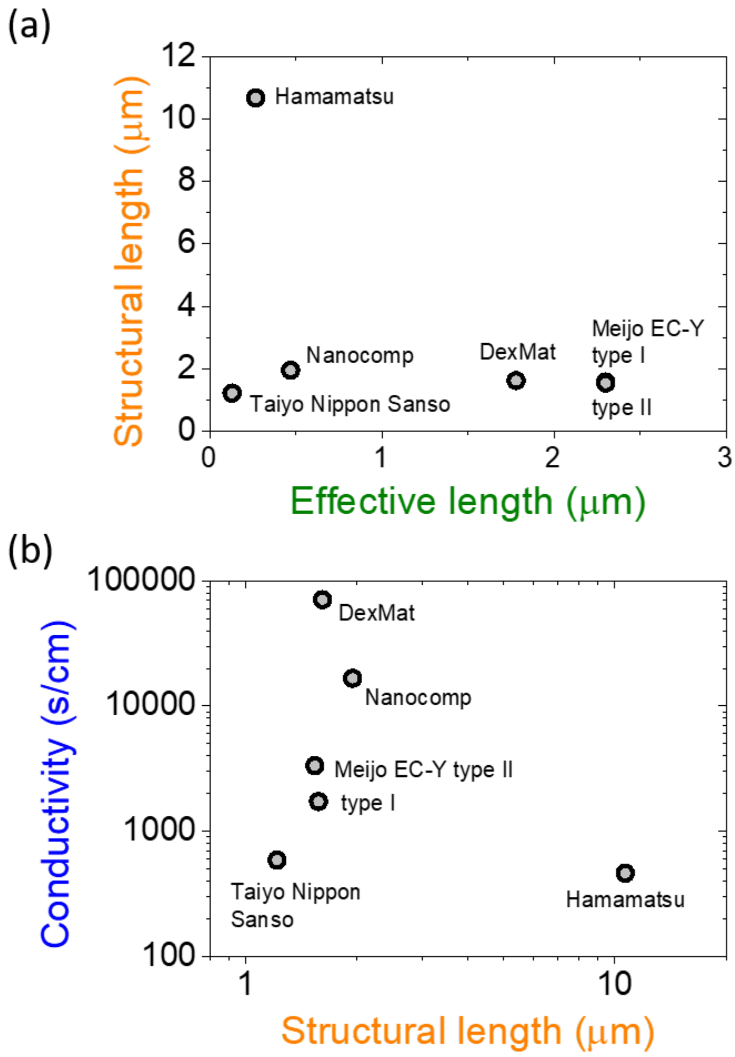

Figure S4. (a) Relationship between structural length and effective length. (b) Structural length dependences of electrical conductivity.

Measurement of CNT-yarn cross section

To calculate the properties of CNT yarns such as density, conductivity, and tensile strength, one factor that may cause a large error is the cross section of the yarn. In this study, we fabricated the fiber diameter measurement system shown in Fig. S5a. The system measures the diameter of a fiber by irradiating it with light (Fig. S5b). It measures the length of the shadow l_{shad} that appears behind the fiber under laser irradiation, and an effective diameter is estimated by averaging the shadow lengths obtained by revolving the sample one time and measuring every 5 degrees. The obtained average diameter is again averaged by measuring more than 300 cross sections in 0.1 mm steps longitudinally.

The obtained yarn diameter d_{yarn}, which represents diameter of each yarn, can be written as,

$$d_{yarn} = \frac{1}{N}\sum\frac{1}{72}\sum_{\theta}^{360°,5°step} l_{shad},$$

where N is the number of observation points. The average yarn cross section $A_{cs}\left(= \pi d_{yarn}^2/4\right)$ is calculated with d_{yarn}. Cross-sectional areas of all yarn types except for DexMat yarn were calculated with this method. The DexMat cross section was estimated from three SEM images because its diameter was too small to measure with the above method.

To estimate the essential cross section of the yarn, we further corrected for voids. Figure 2 shows a cross-sectional SEM image of Nanocomp Miralon yarn. It is clear that there are nonnegligible voids. These voids were counted with the help of ImageJ software and manual corrections (Fig. 5d)) and their total area was calculated (A_{void}). To correct A_{cs}, a calibration coefficient $\phi_{cs,calib}\left(= \frac{1}{3}\sum_i^3 \left(A_{cs,i} - A_{void,i}\right)/A_{cs,i}\right)$ was used, where three points were measured to reduce the effect of local deviations. Consequently, we used the calibrated yarn cross section, $A_{cs,calib}\left(= \phi_{cs,calib}A_{cs}\right)$ for Nanocomp Miralon yarn in this study.

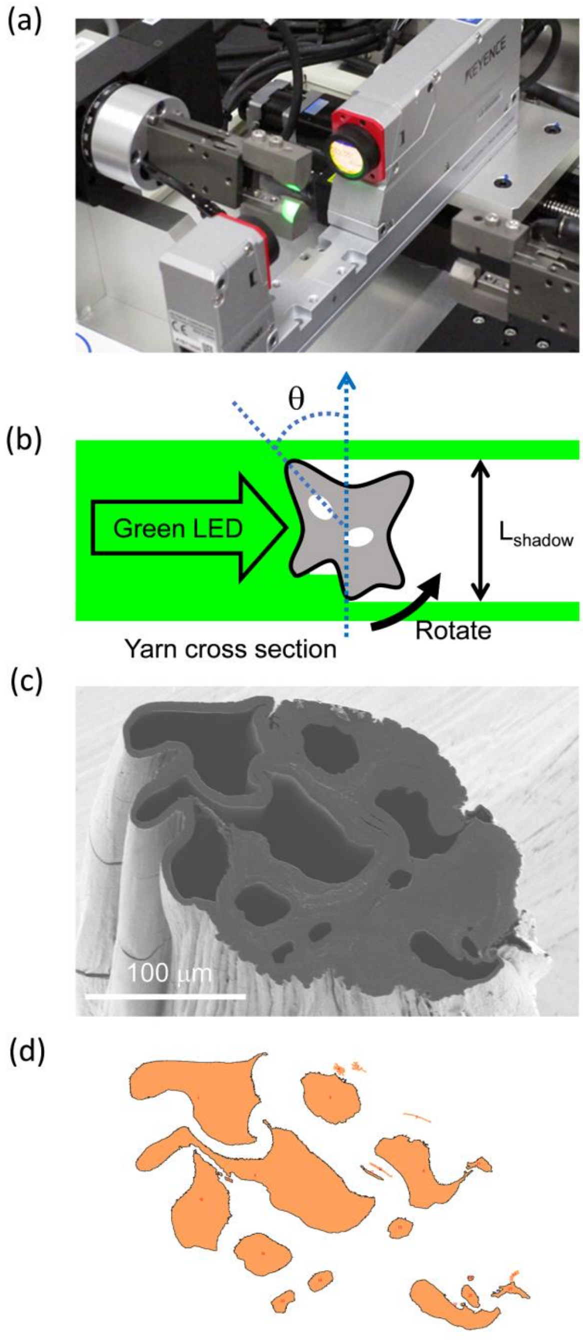

Figure S5. a) Photograph of custom-made fiber diameter measurement system including high-speed optical micrometer (LS-9006MR series, Keyence, Osaka, Japan). b) Schematic illustration of how to measure yarn diameter. c) Cross-sectional SEM image of Nanocomp Miralon yarn. d) Voids extracted from the SEM image by using ImageJ software.

Carbonaceous purity estimation of CNT yarns

Thermogravimetric analysis (TGA) was used to estimate the carbonaceous purity of the CNT yarns. A TGAQ500 (TA instruments, New Castle, DE, USA) was used. Pure oxygen gas was used as the purge gas. The heating rate was controlled in response to the measured weight change. 1 mg of CNT yarn was used for each measurement. For a rough estimate of the CNT ratio in yarn, the weight change between 400°C from 800°C was considered to be the change in the amount of carbonaceous material.[3-6] Because it is not easy to separate CNTs from the other carbonaceous materials like amorphous carbon and graphite in the TGA results, the whole estimated amount of carbonaceous material was treated as the amount of CNT for calculating the related parameters in this study.

Table S1. Carbonaceous content of CNT yarns estimated by TGA.

	Hamamatsu Carbonics	Taiyo Nippon Sanso	Nanocomp Miralon	Meijo EC-Y type I	Meijo EC-Y type II	DexMat
Carbonaceous content estimated by TGA (wt%)	100	100	75	60	60	80

References

1. Morimoto, T. *et al.* Length-Dependent Plasmon Resonance in Single-Walled Carbon Nanotubes. *ACS Nano* **8**, 9897–9904 (2014).
2. Tajima, N. et al., Nanotube-Length and Density Dependences of Electrical and Mechanical Properties of Carbon Nanotube Fibres Made by Wet Spinning, *Carbon*, **152**, 1-6 (2019).
3. Yoshida, H., Sugai, T. & Shinohara, H. Fabrication, purification, and characterization of double-wall carbon nanotubes via pulsed arc discharge. *J. Phys. Chem. C* **112**, 19908–19915 (2008).
4. Park, J. G. *et al.* Effects of surfactants and alignment on the physical properties of single-walled carbon nanotube buckypaper. *J. Appl. Phys.* **106**, 104310 (2009).
5. Zhang, S. *et al.* Ultra-high conductivity and metallic conduction mechanism of scale-up continuous carbon nanotube sheets by mechanical stretching and stable chemical doping. *Carbon N. Y.* **125**, 649–658 (2017).
6. Paukner, C. & Koziol, K. K. K. Ultra-pure single wall carbon nanotube fibres continuously spun without promoter. *Sci. Rep.* **4**, 1–7 (2014).
